# Clinical Profile and Admission Outcomes of Patients With Acute Pulmonary Embolism at a Resource‐Constrained Hospital in Ghana: A Retrospective Pilot Study

**DOI:** 10.1002/hsr2.71951

**Published:** 2026-03-02

**Authors:** Prosper Adjei, Samuel Kyeremeh Adjei, Kingsley Owusu Manu

**Affiliations:** ^1^ Department of Internal Medicine Methodist Hospital Wenchi Ghana

**Keywords:** acute pulmonary embolism, anticoagulation, clinical profile, computed tomography pulmonary angiography, outcomes

## Abstract

**Background and Aims:**

Pulmonary embolism is a cardiovascular disease associated with significant morbidity and mortality. There is a paucity of literature regarding this condition from the Ghanaian perspective. This study aimed at evaluating the clinical profile and admission outcomes of patients with acute pulmonary embolism at the Methodist Hospital, Wenchi, Ghana.

**Methods:**

A retrospective pilot study was conducted by analyzing medical records of patients diagnosed with acute pulmonary embolism from September 2023 to March 2025. Data on demographics, clinical manifestations, risk factors, investigations, treatment, and outcomes of admission were collected.

**Results:**

Out of 17 patients enrolled in the study, 11 (64.7%) were females. The mean age was 58.8 ± 13.0 years. The commonest risk factors were age > 65 years (*n* = 6), immobilization (*n* = 5), obesity (*n* = 4), and recent surgery (*n* = 3). Hypertension and diabetes mellitus were the predominant comorbidities. Dyspnea (76.5%, *n* = 13) and chest pain (*n* = 5) were the most frequent presenting symptoms while tachypnea (70.6%, *n* = 12), tachycardia (70.6%, *n* = 12), and hypoxia (52.9%, *n* = 9) were the commonest clinical signs. Three patients had hemodynamic instability. Pretest probability assessment was infrequently performed (*n* = 7). Sinus tachycardia (76.5%, *n* = 13) was the predominant electrocardiographic abnormality. The majority (58.8%, *n* = 10) had intermediate‐risk pulmonary embolism whereas 3 patients were classified as having high‐risk pulmonary embolism. Two in‐hospital mortalities were recorded.

**Conclusion:**

Clinical features and predisposing factors were largely similar to those described in the literature. Also, pretest probability assessment was underutilized. In‐hospital mortality rate (11.8%) was relatively high. Large‐scale, multicenter prospective studies are recommended to assess long‐term complications of acute pulmonary embolism such as recurrence and chronic thromboembolic pulmonary hypertension.

**Abbreviations**
COPD: chronic obstructive pulmonary disease
CTPA: computed tomography pulmonary angiography
CUS: compression ultrasonography
DVT: deep vein thrombosis
ECG: electrocardiogram
ESC: European Society of Cardiology
NOACs: novel oral anticoagulants
PE: pulmonary embolism
PESI: Pulmonary Embolism Severity Index
SD: standard deviation
sPESI: Simplified Pulmonary Embolism Severity Index
TTE: transthoracic echocardiography
VKAs: vitamin K antagonists
VTE: venous thromboembolism

## 1 Introduction

Venous thromboembolism (VTE) is characterized by the formation of blood clots in the veins. It is regarded as a continuum which comprises deep vein thrombosis (DVT) and pulmonary embolism (PE) [1]. PE is a life‐threatening condition that results from the partial or complete occlusion of the pulmonary artery or its branches by thrombus which migrates from the venous circulation to the pulmonary vasculature [2, 3]. More often than not, it originates from DVT in the lower extremities. The occurrence of PE hinders efficient gaseous exchange by reducing blood flow in the pulmonary circulation, leading to ventilation–perfusion mismatch and ultimately causing hypoxemia [4]. Also, PE increases right ventricular afterload through mechanical obstruction of the pulmonary vasculature. The release of vasoactive mediators such as serotonin and thromboxane A2 from activated platelets results in pulmonary vasoconstriction which further increases the right ventricular afterload. This may lead to right ventricular failure and circulatory collapse [5].

Globally, PE ranks as the third leading cause of cardiovascular death after myocardial infarction and stroke [6, 7]. While the actual worldwide incidence of PE remains unknown, an estimated incidence rate of 117/100,000 population has been reported in the United States with 600,000 cases of VTE diagnosed annually [3]. About 300,000 deaths/year in the United States are attributable to PE. A study conducted in six European countries with a population of 454.4 million found that VTE was responsible for more than 370,000 deaths [8]. The economic burden imposed by VTE is substantial. The medical treatment of 375,000–425,000 newly diagnosed VTE cases is conservatively estimated to cost the United States healthcare system $7–$10 billion yearly [9]. Due to the lack of reliable, large‐scale data relating to PE on the continent, African populations are highly underrepresented in global health reports. Some studies have reported the prevalence of PE in sub‐Saharan Africa to be 1.4%–7% [10]. Comprehensive African‐specific data are needed to understand the actual burden of the disease, identify region‐specific risk factors and to guide the efficient use of limited advanced diagnostic tools.

The clinical manifestations of acute PE are highly variable and nonspecific. Affected individuals commonly present with dyspnea, chest pain, cough, syncope, or hemoptysis. In some instances, patients may be asymptomatic [4, 11]. The nonspecificity of the clinical features makes acute PE a “great masquerader” which poses significant diagnostic challenge to clinicians. An autopsy‐based study conducted at a tertiary hospital in Ghana revealed that PE was frequently misdiagnosed as other respiratory disorders and infectious diseases [12].

Computed tomography pulmonary angiography (CTPA) is the gold standard diagnostic tool for PE [4, 10, 13]. However, access to this imaging technique is limited in most African countries as a result of high costs, limited availability of computed tomography scanners and inadequate trained personnel to man the equipment. This worrying situation coupled with the nonspecific nature of the clinical features may result in delayed or misdiagnosis of PE which can lead to significant morbidity and mortality.

The clinical features and risk factors for PE have been widely studied in Europe, North America, and the Far East [14]. There is, however, a dearth of literature from the Ghanaian context in this area. This study sought to evaluate the clinical characteristics, risk factors, treatment, and outcomes of patients with acute PE who were admitted at the Methodist Hospital, Wenchi, Ghana.

## 2 Materials and Methods

### 2.1 Study Design and Setting

This was a descriptive pilot study conducted by retrospectively analyzing medical records of patients at the Internal Medicine Department of the Methodist Hospital, Wenchi, Ghana from September 2023 to March 2025. Approval and waiver of informed consent were granted by the Research Ethics Committee of the Methodist Hospital (Ethics Approval Reference Number: MHW/INT/56) before commencement of the study.

The Methodist Hospital is a 250‐bed capacity health facility located in Wenchi in the Bono Region of Ghana. It provides comprehensive primary healthcare as well as specialist services in internal medicine, pediatrics, obstetrics and gynecology, urology, general surgery, and orthopedics. It serves as the main referral center for the Wenchi Municipality and its environs.

### 2.2 Study Population

**Figure 1:**
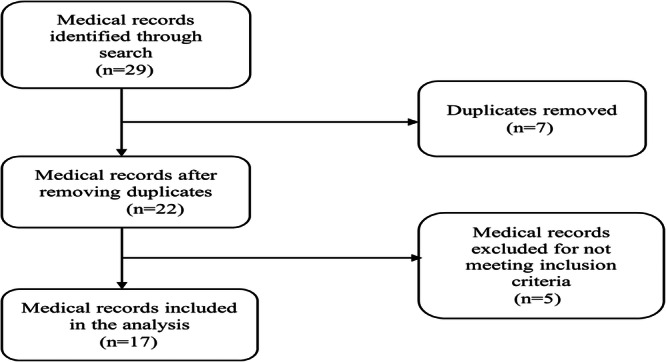
Flowchart of patient inclusion and exclusion.

All patients with suspected PE who were admitted to the Internal Medicine Department of the Methodist Hospital during the study period were referred to another hospital for CTPA. Only patients with CTPA‐confirmed acute PE were included in the analysis. Patients with suspected PE whose medical records had no documentation of CTPA findings as well as those with angiographic evidence of chronic PE were excluded from the study. Also, individuals with a previous history of PE who were hospitalized for other medical reasons were excluded from the current study (Figure 1).

### 2.3 Data Collection

A thorough search was conducted on the electronic health information management system of our hospital using the International Classification of Diseases, 10th Revision (ICD‐10) Codes I26.0 (pulmonary embolism with mention of acute cor pulmonale), I26.9 (pulmonary embolism without mention of acute cor pulmonale), 126.02 (saddle embolus of pulmonary artery with acute cor pulmonale), 126.92 (saddle embolus of pulmonary artery without acute cor pulmonale), 126.93 (single subsegmental thrombotic pulmonary embolism without acute cor pulmonale), and 126.94 (multiple subsegmental thrombotic pulmonary emboli without acute cor pulmonale). The medical records of the identified patients were carefully scrutinized for confirmation of the diagnosis of acute PE. Data relating to demographics, clinical manifestations, risk factors, comorbidities, investigations, treatment, and outcomes of admission were extracted from the electronic medical records of the eligible patients. The retrieved data were entered into a Microsoft Excel spreadsheet.

### 2.4 Study Outcomes

This study sought to track two main admission outcomes of patients with acute PE: *alive* or *death from any cause during hospitalization*.

### 2.5 Definitions

- Central PE: It refers to PE that occurs in the pulmonary trunk, left and right main pulmonary arteries, interlobar arteries, or lobar arteries [14].
- Peripheral PE: It is defined as PE occurring in the segmental or subsegmental arteries [14].
- Hemodynamic instability: It is characterized by systolic blood pressure of < 90 mmHg or systolic blood pressure drop ≥ 40 mmHg, lasting > 15 min and not caused by new‐onset arrhythmia, hypovolemia, or sepsis [11].
- High‐risk PE: It refers to PE that is characterized by hemodynamic instability.
- Intermediate‐risk PE: It is the type of PE that occurs in hemodynamically stable patients with Simplified Pulmonary Embolism Severity Index (sPESI) ≥ 1.
- Low‐risk PE: It is defined as PE occurring in hemodynamically stable patients with sPESI of zero.
- Hypoxia: Peripheral oxygen saturation (SpO_2_) < 90% on room air [15].
- Immobilization: Bed rest or bedridden for > 3 days in the month prior to the diagnosis of PE [16].
- Recent surgery: Any surgical procedure performed under regional or general anesthesia in the 4 weeks preceding the diagnosis of PE [14].
- Recent long‐distance travel: Any continuous journey lasting > 4 h within the 4 weeks preceding the diagnosis of PE [16].
- Positive D‐dimer test: D‐dimer level > 0.5 mg/L (500 ng/mL).
- Tachycardia: Pulse rate > 100 beats/min.
- Tachypnea: Respiratory rate > 20 cycles/min.

### 2.6 Statistical Analysis

Data analysis was performed using Statistical Package for Social Sciences (SPSS) Version 25. Continuous variables were expressed as mean ± standard deviation while categorical variables were presented as frequencies and percentages. *χ* ^2^ test was used to compare categorical variables between survivors and nonsurvivors of acute PE during hospitalization. A *p* < 0.05 was considered statistically significant.

## 3 Results

### 3.1 Sociodemographic Characteristics of the Patients

A total of 29 medical records were identified through a search on the electronic health information management system out of which 7 duplicates were removed. Five medical records were excluded for not satisfying the inclusion criteria. The medical records of 17 patients who were diagnosed with acute PE following CTPA over the study period were included in the final analysis. Eleven (64.7%) of the enrolled patients were females. The average age of the patients was 58.8 ± 13.0 years with a range of 40–82 years. More than one‐third (*n* = 6) of them were older than 65 years. Table 1 summarizes the demographic characteristics of the patients.

**Table 1:** Age and gender distribution of patients with acute pulmonary embolism.

Characteristic	Frequency (*n*)	Percentage (%)
Age (years)		
Mean ± SD	58.8 ± 13.0	
Range	40–82	
Age group		
40–45	3	17.6
46–50	3	17.6
51–55	2	11.8
56–60	1	5.9
61–65	2	11.8
> 65	6	35.3
Gender		
Male	6	35.3
Female	11	64.7

Abbreviation: SD, standard deviation.

### 3.2 Predisposing Factors and Comorbidities in Patients With Acute Pulmonary Embolism

The majority (82.4%, *n* = 14) of the patients had at least one predisposing factor for acute PE. The predominant predisposing factors were age > 65 years (*n* = 6), immobilization (*n* = 5), obesity (*n* = 4), and recent surgery (*n* = 3). Hypertension (*n* = 9) was the commonest comorbidity followed by diabetes mellitus (*n* = 4). Notably, 3 patients had no comorbidities or identifiable risk factors for acute PE. Table 2 itemizes the predisposing factors and comorbidities identified in this study.

**Table 2:** Predisposing factors and comorbidities in patients with acute pulmonary embolism.

Predisposing factors and comorbidities	Frequency (*n*)	Percentage (%)
Age > 65 years	6	35.3
Immobilization	5	29.4
Hypertension	9	52.9
Diabetes mellitus	4	23.5
Recent surgery	3	17.6
Congestive heart failure	1	5.9
Obesity	4	23.5
Long‐distance travel	1	5.9
COPD	1	5.9
No predisposing factor or comorbidity	3	17.6

Abbreviation: COPD, chronic obstructive pulmonary disease.

### 3.3 Clinical Features and Pretest Probability Assessment

Dyspnea (76.5%, *n* = 13) and chest pain (29.4%, *n* = 5) were the most frequently reported symptoms among the patients. Less commonly reported symptoms were syncope, cough, hemoptysis, and easy fatigue. The average duration of symptom onset to hospitalization was 2.2 days, with more than half (58.8%, *n* = 10) of the patients presenting to the hospital within 24 h of symptom onset. The most commonly identified clinical signs included tachypnea (70.6%, *n* = 12), tachycardia (70.6%, *n* = 12), and hypoxia (52.9%, *n* = 9). Three patients had hemodynamic instability at presentation. None of them had signs of lower extremity DVT.

**Figure 2:**
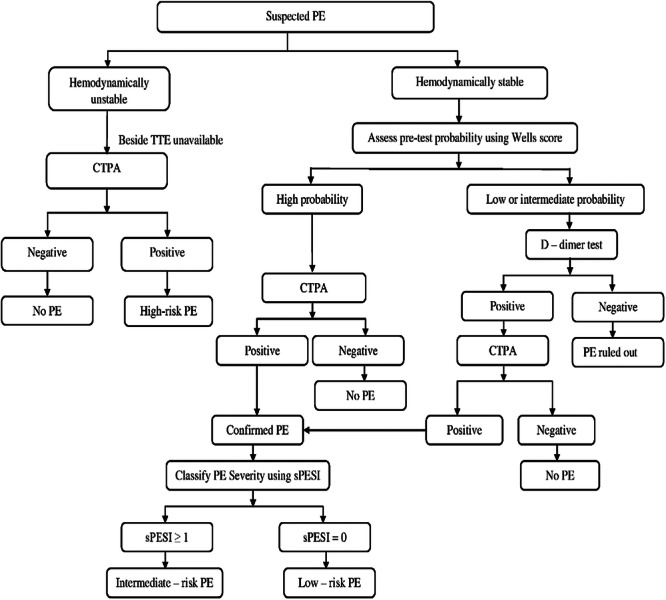
Diagnostic algorithm and classification of acute PE severity at the Methodist Hospital, Wenchi, Ghana. CTPA, computed tomography pulmonary angiography; PE, pulmonary embolism; TTE, transthoracic echocardiography.

There was documentation of pretest probability assessment for only 7 patients using the 3‐level Wells score. Out of this, 6 individuals were considered to be at intermediate risk for PE, whereas 1 patient had a high pretest probability score (Figure 2). The clinical features and pretest probability assessment of the patients are captured in Table 3.

**Table 3:** Clinical manifestations and pretest probability assessment of patients with acute pulmonary embolism.

Characteristic	Frequency (*n*)	Percentage (%)
Symptom		
Dyspnea	13	76.5
Chest pain	5	29.4
Syncope	3	17.6
Cough	2	11.8
Hemoptysis	1	5.9
Easy fatigue	1	5.9
Symptom duration		
1 day	10	58.8
2–5 days	6	35.3
> 5 days	1	5.9
Clinical sign		
Hypoxia	9	52.9
Tachypnea	12	70.6
Tachycardia	12	70.6
Hemodynamic instability	3	17.6
Signs of lower extremity DVT	0	0.0
3‐level Wells score (*n* = 7)		
< 2 points	0	0.0
2–6 points	6	85.7
> 6 points	1	14.3

Abbreviation: DVT, deep vein thrombosis.

### 3.4 Diagnostic Tests, Severity Assessment, and Risk Stratification of Acute Pulmonary Embolism

**Figure 3:**
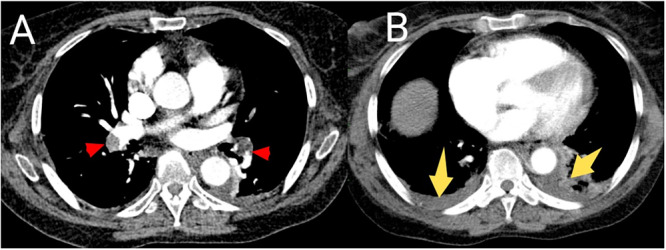
Axial CTPA images in mediastinal window showing filling defects in the right and left pulmonary arteries (red arrows in (A)) and minimal bilateral pleural effusions (yellow arrows in (B)) in a 74‐year‐old hypertensive and diabetic patient, presenting with sudden‐onset dyspnea and chest pain.

**Figure 4:**
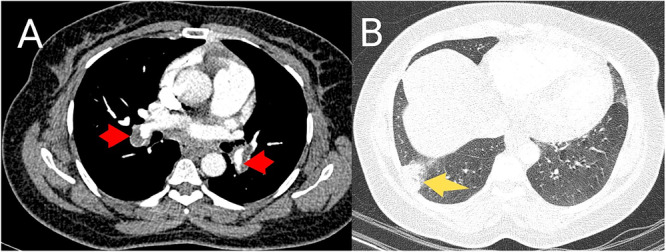
Axial CTPA images in mediastinal (A) and lung (B) windows, from a 43‐year‐old female presenting with chest pain on the third day after total abdominal hysterectomy, demonstrating bilateral filling defects in the right and left pulmonary arteries (red arrows in (A)) as well as peripheral, wedge‐shaped opacity (yellow arrow in (B)) suggestive of pulmonary infarction in the right lower lobe.

Electrocardiogram (ECG) was done in all the patients, out of which 13 (76.5%) had sinus tachycardia. Other notable electrocardiographic findings were S1Q3T3 (*n* = 2), T wave inversion in leads V1–V4 (*n* = 2), and normal sinus rhythm (*n* = 2). Chest X‐ray was performed in 13 (76.5%) patients with most (46.2%, *n* = 6) of them demonstrating normal findings. Four patients had pleural effusion while three had wedge‐shaped opacity suggestive of pulmonary infarction. CTPA was performed in all the patients. With respect to anatomic location, 12 (70.6%) people had angiographic evidence of thrombi in both central and peripheral pulmonary arteries. Four individuals had central PE only and one person had thrombi localized to the peripheral pulmonary arteries alone. There was bilateral lung involvement in 82.4% (*n* = 14) of the patients (Figures 3 and 4). D‐dimer testing was done in 14 cases with all of them showing positive results. The average D‐dimer level was 7.2 mg/L.

Regarding the assessment of PE severity and the risk of early death, 76.5% (*n* = 13) of the patients had sPESI ≥ 1, while the rest had sPESI of zero. The number of cases categorized as high‐risk PE, intermediate‐risk PE, and low‐risk PE were 3 (17.6%), 10 (58.8%), and 4 (23.5%), respectively. The findings of the various diagnostic tests and other details relating to risk stratification of the patients are summarized in Table 4.

**Table 4:** Diagnostic tests, risk stratification, and severity classification of patients with acute pulmonary embolism.

Investigation	Frequency (*n*)	Percentage (%)
ECG findings		
Sinus tachycardia	13	76.5
S1Q3T3	2	11.8
T wave inversion in V1–V4	2	11.8
Right atrial enlargement	1	5.9
Normal	2	11.8
Chest X‐ray findings (*n* = 13)		
Normal	6	46.2
Wedge‐shaped opacity	3	23.0
Pleural effusion	4	30.8
PE location on CTPA		
Central	4	23.5
Peripheral	1	5.9
Both central and peripheral	12	70.6
Lung involved on CTPA		
Right‐sided	3	17.6
Bilateral	14	82.4
D‐dimer (*n* = 14)		
Positive	14	100
sPESI		
Low risk (0 point)	4	23.5
High risk (≥ 1 point(s))	13	76.5
PE severity classification		
High‐risk PE	3	17.6
Intermediate‐risk PE	10	58.8
Low‐risk PE	4	23.5

Abbreviations: ECG, electrocardiogram; PE, pulmonary embolism; CTPA, computed tomography pulmonary angiography; sPESI, Simplified Pulmonary Embolism Severity Index.

### 3.5 Treatment and Admission Outcomes

For initial anticoagulation, a substantial majority (94.1%, *n* = 16) of the patients were given subcutaneous enoxaparin, with only 1 person receiving rivaroxaban. With regard to maintenance anticoagulant therapy, 16 patients received rivaroxaban while warfarin was prescribed for 1 person. Inotropic support was provided for 3 patients with hemodynamic instability. A total of 15 (88.2%) patients survived during admission. In total, 2 patients succumbed to their illness, giving an in‐hospital mortality rate of 11.8% (Table 5).

**Table 5:** Treatment and admission outcomes of patients with acute pulmonary embolism.

Characteristic	Frequency (*n*)	Percentage (%)
Initial anticoagulant		
Enoxaparin	16	94.1
Rivaroxaban	1	5.9
Maintenance anticoagulant		
Rivaroxaban	16	94.1
Warfarin	1	5.9
Supportive treatment		
Inotrope (norepinephrine)	3	17.6
Admission outcome		
Alive	15	88.2
Dead	2	11.8

### 3.6 Comparison of Survivors and Nonsurvivors of Acute Pulmonary Embolism

*χ* ^2^ test was performed to examine the relationship between clinical variables and admission outcomes of patients with acute PE. The analysis indicated that there was no statistically significant association between any of the clinical variables and admission outcomes (Table 6).

**Table 6:** Chi‐square analysis to assess the association between clinical variables and admission outcomes of patients with acute pulmonary embolism.

Variable	Outcome		*p*
	Alive (*n* = 15)	Dead (*n* = 2)	
Hemodynamic status			0.201
Stable	13 (92.9)	1 (7.1)	
Unstable	2 (66.7)	1 (33.3)	
Hypoxia			0.929
Yes	8 (88.9)	1 (11.1)	
No	7 (87.5)	1 (12.5)	
Tachypnea			0.331
Yes	10 (83.3)	2 (16.7)	
No	5 (100)	0 (0.0)	
Tachycardia			0.331
Yes	10 (83.3)	2 (16.7)	
No	5 (100)	0 (0.0)	
PE location on CTPA			0.624
Central	14 (87.5)	2 (12.5)	
Peripheral only	1 (100)	0 (0.0)	
Lung involved on CTPA			0.486
Unilateral (right‐sided)	3 (100)	0 (0.0)	
Bilateral	12 (85.7)	2 (14.3)	
sPESI			0.404
High risk (≥ 1 point(s))	11 (84.6)	2 (15.4)	
Low risk (0 point)	4 (100)	0 (0.0)	

Abbreviations: CTPA, computed tomography pulmonary angiography; PE, pulmonary embolism; sPESI, Simplified Pulmonary Embolism Severity Index.

## 4 Discussion

PE is a common condition which remains a major public health concern globally. Timely diagnosis and prompt initiation of appropriate treatment are crucial for preventing PE‐related mortality [4]. Unfortunately, the diagnosis of this cardiovascular disease in resource‐constrained settings is fraught with challenges. To the best of our knowledge, this is the first study conducted in Ghana to evaluate the clinical profile and admission outcomes of patients with acute PE.

The majority (64.7%, *n* = 11) of patients enrolled in this study were females which aligns with observations from several earlier studies [17, 18, 19, 20]. Other researchers have reported a male preponderance [16, 21, 22, 23]. While gender per se is not a direct cause of PE, there may be differences in risk factors and clinical presentation between men and women [24, 25]. An appreciation of these gender‐specific nuances is critical for the diagnosis and effective management of PE. The mean age of patients in our study was 58.8 ± 13.0 years which is comparable to the average age of participants reported in previous studies [7, 19, 26]. As opposed to our analysis, studies carried out in the Democratic Republic of Congo [18], Sierra Leone [27], and Thailand [28] reported a higher average age, while others conducted in Nepal [20] and Qatar [29] revealed a lower mean age of participants. The variations may be explained by differences in study settings and demographic features of participants. Consistent with existing literature [30], patients aged > 65 years constituted the majority of our study population. It is estimated that over 60% of VTE events occur in persons aged ≥ 65 years. Additionally, these individuals tend to have a higher VTE‐related morbidity and mortality compared to younger patients [30].

A wide range of factors can increase an individual's risk of developing acute PE. In this research, 82.4% (*n* = 14) of the patients had at least one predisposing factor for acute PE. This closely mirrors findings of similar studies conducted in Saudi Arabia (83.5%) [14] and Thailand (80.5%) [19]. The commonest risk factor was age > 65 years. A systematic review involving 7650 patients from 10 African countries found age > 65 years to be a major risk factor for acute PE [13]. Age‐related physiological changes, including elevated fibrinogen and reduced level of antithrombin‐3, create a more hypercoagulable state in older people. This prothrombotic state in combination with high prevalence of chronic diseases and increased likelihood of prolonged immobilization, puts elderly individuals at a higher risk of developing PE [31]. In line with several prior studies [13, 16, 19, 29], our analysis also identified immobilization, obesity, and recent surgery as common risk factors for acute PE. Similarities in clinical characteristics of study participants may be responsible for this. Regarding comorbidities, hypertension and diabetes mellitus were the most frequently identified comorbid conditions. This finding corroborates the outcomes of previously published studies [16, 18, 23, 29]. In diabetic patients, hyperglycemia can cause endothelial dysfunction. It can also increase coagulation activation and impair fibrinolysis. These changes can lead to a prothrombotic state which may result in VTE [32]. Equally, hypertension has been shown to be strongly associated with PE [33]. It is, therefore, crucial to maintain adequate glycemic and blood pressure control in order to minimize the risk of PE in affected patients.

The clinical manifestations of acute PE are diverse and nonspecific [4, 11]. Just like many other studies [14, 18, 20, 21, 23], the current analysis identified dyspnea and chest pain as the most commonly reported symptoms. A study conducted at two large tertiary hospitals in Ghana discovered that PE was the culprit in 14.9% of patients presenting with acute chest pain [34]. These findings underscore the need for meticulous clinical assessment, risk factor evaluation, and appropriate investigations to rule out PE in instances where the underlying etiologies of dyspnea and chest pain are not immediately apparent. With respect to clinical signs, the majority of our patients had tachypnea (70.6%, *n* = 12), tachycardia (70.6%, *n* = 12), and hypoxia (52.9%, *n* = 9). These observations are consistent with current literature [17, 19, 29]. Furthermore, three patients in this study satisfied the criteria for hemodynamic instability which characterizes acute high‐risk PE [11]. Echocardiographic examination is recommended in individuals with suspected high‐risk PE as the absence of right ventricular dysfunction essentially rules out PE as the cause of hemodynamic instability [11]. Echocardiographic evaluation, however, could not be performed for these three patients during admission because this diagnostic service is not readily available in our hospital or any of the other facilities in our region. Interestingly, none of the patients had signs of lower extremity DVT such as swelling or tenderness. A research conducted at the National Cardiothoracic Center in Accra, Ghana to determine the outcome of thrombolysis for massive PE found that only one person out of 17 patients had a history of calf pain prior to the onset of symptoms of PE, but there was no lower limb swelling or tenderness in any of the patients [35]. It must be emphasized that the absence of signs does not exclude the possibility of lower extremity DVT. Although PE can occur in isolation [36], the majority of cases originate from DVT in the lower extremities [4, 11]. Compression ultrasonography (CUS) can detect DVT in 30%–50% of patients with PE [11]. Despite its usefulness, a normal bilateral proximal venous ultrasound scan does not rule out PE. When there is objective evidence of PE in a patient without signs of lower extremity DVT, the PE may have originated from pelvic veins or embolized completely from a lower extremity vein [36]. Given the economic circumstances of the patients and the high out‐of‐pocket costs involved in transporting them to another facility for CTPA and CUS, it was necessary to prioritize the former since it is the most preferred diagnostic tool for PE. In view of this, CUS was not performed for any of the patients in this study.

The assessment of pretest probability is an important step in the evaluation of patients with suspected PE [4, 11]. In this study, pretest probability assessment using the 3‐level Wells score was documented in only seven patients. Similar to an earlier research conducted in Sierra Leone [27], this result highlights the underutilization of pretest probability in the evaluation of patients with suspected PE. The reliance of clinicians on their clinical judgment and experience in requesting for CTPA may have contributed to this. It is important for clinicians to incorporate pretest probability assessment in their practice to avoid unnecessary diagnostic tests. Minimizing unnecessary investigations can translate into significant cost savings for patients and healthcare systems, especially in resource‐deprived areas.

The predominant electrocardiographic abnormality in this cohort of patients was sinus tachycardia (76.5%, *n* = 13) which is in consonance with existing literature [16, 20, 22]. The classic S1Q3T3 pattern (*n* = 2) was infrequently encountered. The primary role of chest X‐ray in the clinical assessment of patients with suspected acute PE is to exclude conditions that mimic PE [2]. Chest X‐ray was normal in most of our patients with the commonest abnormality being pleural effusion (*n* = 4). D‐dimer is a product of fibrin degradation and raised plasma levels reflect ongoing activation of coagulation and fibrinolysis in patients with acute thrombosis. In this analysis, D‐dimer levels were elevated in all patients for whom testing was done. The definitive diagnosis of acute PE was made by performing CTPA in all the patients. The majority (70.6%, *n* = 12) of them had angiographic evidence of thrombi in both central and peripheral pulmonary arteries. This was followed by localization of thrombi to the central pulmonary arteries alone in four cases. One person had clots localized exclusively to the peripheral pulmonary arteries. Again, there was bilateral lung involvement in 82.4% (*n* = 14) of the patients. The site of thromboembolic occlusion has emerged as a debatable prognostic factor in acute PE. Alonso Martinez and colleagues found that individuals with central PE had an all‐cause mortality rate of 40% while those with peripheral PE had an overall mortality of 27% [37]. In stark contrast, a study carried out in Saudi Arabia reported similar all‐cause mortality rates in patients with central and peripheral PE (i.e., 5.3% vs. 6.6%; *p* = 0.61) [38]. Our analysis did not show any statistically significant difference between survivors and nonsurvivors of acute PE with regard to thrombus location. In the light of these conflicting reports, further research is required to ascertain the true impact of thrombus location on the survival of patients with acute PE.

Risk stratification and classification of PE severity are prerequisites for determining the appropriate therapeutic interventions for patients with acute PE. The original Pulmonary Embolism Severity Index (PESI) and its simplified version (sPESI) are validated tools for assessing the risk of 30‐day mortality in patients with acute PE [11, 39]. In our analysis, the sPESI was used. Thirteen (76.5%) patients had sPESI ≥ 1, indicating a high risk of 30‐day mortality. The rest had a low risk of 30‐day mortality with sPESI of zero. In this cohort of patients, the determination of PE severity was based on the presence of hemodynamic instability and sPESI. Most (58.8%, *n* = 10) of them had intermediate‐risk PE, with the least number (*n* = 3) of cases categorized as high‐risk PE. This finding is similar to that of a research conducted in South Africa [17]. It is, however, at variance with studies carried out in India [21] and Angola [23] which reported the highest number of cases in the low‐risk category. The discrepancies in PE severity classification between the current study and the previous ones may be due to differences in clinical characteristics of the patients as well as the inclusion of echocardiographic findings in the previous studies.

With the exception of one patient who received rivaroxaban right from the outset, all the others were given subcutaneous enoxaparin for initial anticoagulation. Subsequently, 15 patients who were started on subcutaneous enoxaparin were transitioned to rivaroxaban for maintenance anticoagulation. After overlapping subcutaneous enoxaparin and warfarin for 5 days to achieve therapeutic international normalized ratio, one person was transitioned to warfarin for maintenance anticoagulation. In patients with suspected PE, particularly those with intermediate or high pretest probability, it is strongly advised to start anticoagulation while awaiting the results of investigations. As recommended by the European Society of Cardiology (ESC), the initiation of parenteral anticoagulation with low‐molecular‐weight heparin or fondaparinux is preferred to unfractionated heparin in most cases [11]. Again, for patients with PE who are eligible for novel oral anticoagulants (NOACs), these agents are recommended over vitamin K antagonists (VKAs) [11]. NOACs have predictable bioavailability and pharmacokinetics, and so they can be given at fixed doses without the need for regular laboratory monitoring. Additionally, NOACs, unlike VKAs, have fewer interactions when administered concurrently with other drugs [11]. For these reasons, the majority of our patients received subcutaneous enoxaparin and rivaroxaban for initial and maintenance anticoagulation, respectively.

Reperfusion therapy rapidly restores blood flow to the lungs and improves right ventricular function in high‐risk PE. Systemic thrombolysis is the recommended first‐line treatment for this type of PE. In instances where this therapeutic approach is contraindicated or has failed, surgical embolectomy or percutaneous catheter‐directed treatment is indicated [11]. Due to the lack of logistics and trained personnel to safely carry out systemic thrombolysis in our hospital, the need for referral to a tertiary center was discussed with the three patients in this study who were categorized as having high‐risk PE. They, however, declined referral on account of financial constraints. Under the circumstances, they were initially treated with subcutaneous enoxaparin and inotropic support was provided by administering norepinephrine. The ESC recommends the use of norepinephrine and dobutamine for the treatment of right ventricular failure in acute high‐risk PE [11].

In total, 15 (88.2%) patients survived during admission while 2 of them unfortunately died, giving an in‐hospital mortality rate of 11.8% which is lower than the rates reported in Cameroon (18.4%) [9], Democratic Republic of Congo (32.6%) [18], and Angola (20%) [23]. The mortality rate found in our study is higher than rates described in studies conducted in Nigeria (9.7%) [26] and Qatar (3.4%) [29]. Differences in clinical characteristics of patients as well as PE severity may account for the variations in in‐hospital mortality rates.

Quite clearly, the diagnostic and management challenges highlighted in this study have health policy implications for Ghana as well as other similar low‐resource settings. To ensure equitable access to quality healthcare, it is incumbent on governments and hospital managers to invest in diagnostic infrastructure in order to enhance early detection and prompt treatment of sinister conditions like PE. As a matter of priority, the Ministry of Health and other relevant agencies should provide the tertiary, regional, and selected district hospitals with the necessary logistics and trained personnel to undertake systemic thrombolysis for patients with PE when indicated. Given the inefficient referral system as well as the limited number of specialists in regional and district hospitals, a successful integration of telemedicine into the existing Ghanaian healthcare system can substantially improve access to specialized or expert medical care. This modality can link patients diagnosed with PE in remote areas of Ghana to specialist physicians for prompt and appropriate management.

This study presents some notable strengths. In addition to being the first of its kind to be conducted in Ghana, it provides valuable insights into the clinical characteristics, risk factors, and admission outcomes of patients with acute PE in a resource‐constrained setting. The reliance on CTPA for the definitive diagnosis of acute PE conforms to globally accepted standards of practice. Furthermore, the current analysis provides useful preliminary data that are necessary for designing more robust and larger studies at the national or continental level. Again, the study indirectly brings to light the logistical and human resource constraints that militate against the delivery of quality healthcare services in most primary and secondary health facilities in Ghana. In spite of the above, the study also has several limitations. First, its retrospective nature may have led to biases in data collection and interpretation. Second, the findings may not be generalizable to the larger population due to the small sample size. Regarding PE severity, the absence of echocardiographic findings and cardiac troponin results could have resulted in misclassification of some of the patients. The incidence of DVT in the study population could not be ascertained because CUS was not performed for any of the patients. Lastly, the absence of data relating to the follow‐up of patients limits insights into the long‐term complications of acute PE.

The unavailability of follow‐up data in the current analysis offers an avenue for future research. Large‐scale, multicenter studies and follow‐up registries are recommended to prospectively collect data on recurrence, long‐term complications like chronic thromboembolic pulmonary hypertension and functional limitation, therapeutic complications, long‐term mortality, and to assess post‐PE quality of life.

## 5 Conclusion

Our study revealed that acute PE most commonly occurred in elderly patients. By and large, the clinical features and predisposing factors were similar to those described in the literature. Also, pretest probability assessment was underutilized. Additionally, in‐hospital mortality rate was relatively high. Large‐scale, multicenter prospective studies are recommended to assess post‐PE quality of life as well as long‐term complications like chronic thromboembolic pulmonary hypertension and recurrence of PE.

## Author Contributions

**Prosper Adjei:** conceptualization, methodology, data curation, formal analysis, writing – original draft, writing – review and editing. **Samuel Kyeremeh Adjei:** data curation, formal analysis. **Kingsley Owusu Manu:** data curation. All authors have read and approved the final version of the manuscript. The corresponding author, Prosper Adjei, had full access to all of the data in this study and takes complete responsibility for the integrity of the data and the accuracy of the data analysis.

## Funding

The authors received no specific funding for this work.

## Ethics Statement

Ethical approval was obtained from the Research Ethics Committee of the Methodist Hospital (Ethics Approval Reference Number: MHW/INT/56) before conducting the study. All data elements were anonymized to protect the privacy of study participants.

## Consent

The need for informed consent was waived by the Research Ethics Committee due to the retrospective nature of the study.

## Conflicts of Interest

The authors declare no conflicts of interest.

## 1 Transparency Statement

The lead author Prosper Adjei affirms that this manuscript is an honest, accurate, and transparent account of the study being reported; that no important aspects of the study have been omitted; and that any discrepancies from the study as planned (and, if relevant, registered) have been explained.

## Data Availability

The data sets that support the findings of this study are available from the corresponding author upon reasonable request.
